# A Homily for Hospital Sunday Reading

**Published:** 1888-06-09

**Authors:** 


### The Hospital

A Weekly Institutional Journal of

Science, Medicine, Nursing, and Philanthropy.

*For Hospitals, Asylums, Medical Practitioners, Students, Nurses, Families, Parishes, Congregations, and the Charitable Public.*

Vol. IV. No. 89.] [June 9, 1888.

### A Homily for Hospital Sunday Reading.

The strength of a motive is a measure of the success of a purpose. Cynics have no faith in a certain class of motives; in their own estimation they are too clever to be imposed upon. If a man strives for objects different from those commonly aimed at by men they will not believe that the animating spirit is different, though they are compelled to admit that the end sought is not the same. The question of motive lies at the root of all benevolent and philanthropic work. Perhaps it would be impossible to find a man who everywhere and always guides his conduct by thoroughly bad motives. On the other hand, it is probably certain that every public man, every statesman, philanthropist, preacher, and teacher, of whatever party, who commands any degree of confidence from any considerable number of his fellow-men, is animated on the whole by feelings and purposes which are not unworthy.

The Kingdom of Benevolence is in a condition of crisis at the present time. Charges of various kinds are made, and evidence is being taken. The public is more or less consciously sitting in judgment. Institutions and their work, men and their motives, are being passed through the slow but searching fire of general inquisition. This process is eminently desirable; but whether desirable or not it is inevitable. The world is often slow, very slow; but it is always sure. Its slowness in arriving at a decision is strictly proportioned to the difficulty and importance of the problem presented for solution. It may decide in an hour that a "Battle of Dorking" is an effective political squib; a century may not be more than sufficient to enable it to fix the place in literature of a "Paradise Lost." Similarly the work of an official or of a single hospital or other charitable institution may be decided upon in a decade, but a whole "Kingdom of Benevolence" must live its life, fulfil its destiny, and then stand or fall by its inherent merit and vitality, by its "fitness" or "unfitness" to "survive."

The foundations of the British charitable system are benevolence and duty; good will and the conviction that the good will must necessarily be translated into good deed. The sense of duty, of duty owed where it is demanded, of duty which will be rewarded and of failure which will be punished, is the mainspring of every kind of permanent and worthy effectiveness in the world. If hospitals and their supporters and officials lose this entirely they will inevitably become feeble, and finally collapse. It is not unlikely that some who read this may attach no concrete importance to it, believing that it is merely the routine utterance of sentiments proper to the occasion and the theme, and is not intended to be taken too seriously. But those who are old enough to have followed the life-history of some of their fellow-men, or who are familiar with that kind of literature which deals with the growth and decadence of states will recognise its practical significance. "As a man thinks in his heart so is he;" and as a nation or a voluntary association is in its ideas, sentiments, and purposes, so does it become in the concrete and the gross; and as it is in the concrete and the gross, so does it appear to its critics, and so is it finally judged, and approved or condemned.

The world is a moral world, a world of reason and of sense. On this all intelligent men and women are now practically agreed, however divergent may be their scientific and philosophic formulæ. Herbert Spencer and the Archbishop of Canterbury, Frederic Harrison and Canon Liddon are alike convinced that morality and duty are distinguishing characteristics of man as man. Hospitals and other charitable institutions must therefore not only be founded on the primary principles of benevolence and duty, but their work in all its departments must be such as not to offend or wound those primary instincts; that work must constantly appeal to those instincts in all classes of human beings; and it must be so obviously good, and broad, and human that those instincts cannot deny the force of the appeal.

Of course no one experienced in human affairs expects any individual man's motives to be so transparent that all the world shall at once see and affirm their purity. Nor is it to be looked for that there will never be any admixture of brass with the pure gold of human benevolence. Man, being what he is, swayed by such passions, surrounded by such environment as he is, will often yield to impulses and pursue objects which cannot be said to coincide with any noble standard of morality. But a practical intelligence takes a wide outlook, and if it sees the general purpose of a man's life to be honest, and, on the whole, worthy, it classes that man among the forces which make for good, and gives him such a degree of encouragement and help as his work at any given period may seem to deserve. It is not to be expected of hospital officials, numbered as they are by tens of thousands, that they will attain generally to a higher standard of motive and action than the average well-conducted human creature. But though no higher than an ordinary standard is to be expected, no lower level must be permitted. Hospital work is essentially a work of benevolence, and it is essential that it be done benevolently. It must be done efficiently as to its results, gently as to its manner, and honestly and economically as to its motives. This the public has a right to demand and to insist upon. Those who cannot conform to this moderate demand should seek another field for their activity than the hospital world.

In this day of crisis and decision at which we have now arrived, we have to ask—have hospitals, on the whole, fulfilled the conditions which benevolent founders and supporters have had a right to expect of them as benevolent institutions? Looking over their history for the past two or three hundred years, what must the answer be? It is eminently, nobly in their favour. To say that there have been no traitors in the camp, no errors of judgment, no failures of duty, would be to state what can never be affirmed with truth of any human being or institution. But a survey of British hospitals for the last three centuries may well make us honestly proud of the good sense, right feeling, and practical capacity of our fellow-countrymen. The record is a record of worthy motive, great purpose, and splendid accomplishment. The best answer to hospital critics is the hospitals themselves and their history.

But having examined, judged, and decided that British hospitals and those who are responsible for them, are of sterling value, and deserving of the confidence of honest men, we should consider it necessary to give practical effect to such a judgment. It seems to us almost as necessary to maintain the hospital system as to maintain the State. Every man contributes his quota to the maintenance of the State by compulsion; every man should also feel himself bound by moral compulsion to do his share towards the support of hospitals. The emphasis in these sentences is to be placed on the words "every man." Therein lies the secret of the future success of hospitals. Hospitals press like a mountain on the energies of the few; they would constitute a burden which would hardly be felt if all men of all ranks would join hands for their defence and advancement. The practical duty of each man whose attention is definitely called to the subject is twofold. He should resolve not only that his own measure of hospital support shall be punctually contributed, but also that he will use all reasonable influence to bring other men to share his convictions and obligations. That being done, the problem of hospitals would be solved.

